# Exploring stakeholders’ perceived problems associated with the care and support of children and youth with mental ill health in Sweden: a qualitative study

**DOI:** 10.1186/s41043-024-00520-8

**Published:** 2024-02-20

**Authors:** Malin Helander, Mio Fredriksson, Malin Lohela-Karlsson

**Affiliations:** 1 Centre for Clinical Research, Västmanland Hospital Västerås, Uppsala University, 721 89 Västerås, Region Västmanland Sweden; 2 Department of Public Health and Caring Sciences, Uppsala University, Uppsala, Sweden

**Keywords:** Collaboration, Inter-organizational, Mental ill health, Stakeholders, Children and youth, Perceived problems

## Abstract

**Background:**

Care and support for children and youth with mental ill health have become more specialized and are provided by an increasing number of stakeholders. As a result, services are often fragmented, inefficient and unco-ordinated, with negative consequences for the service user and their family. Enhanced collaboration could lead to improved care and support but requires a shared understanding and a joint problem formulation between involved stakeholders to commence. The aim of this study was to explore different stakeholders’ perceived problems associated with delivering care and support to children and youth with mental ill health and to discuss how the perceived problems relate to collaboration.

**Methods:**

A qualitative descriptive study was conducted, using short statements of perceived problems written by stakeholders involved in the care and support of children and youth with mental ill health during an inter-organizational workshop. The 26 stakeholders represented school and student health, primary health care, specialist care, social services, and different service user organizations. Data were collected during February 2020. Inductive content analysis with a summative approach was used when analysing the data.

**Results:**

The perceived problems were summarized in a model consisting of four main categories: ***Resources and governance; Collaboration and co-ordination; Knowledge and competence; and Stigma and confidence***, containing 24 subcategories. These categories and subcategories were distributed over three levels: *Societal level, Organizational level* and *Individual level.* The perceived problems were shared on the category level but to some extent varied between stakeholder groups on the subcategory level. The perceived problems were either directly or indirectly related to collaboration.

**Conclusions:**

The perceived problems often acted as barriers to achieving successful collaboration. The problems were distributed on all three levels in the developed model, indicating a complex problem. Even though the perceived problems were shared by stakeholders on an overall level, the findings indicate that the stakeholders did not have a completely shared understanding of the perceived problems, as they tended to focus on aspects most relevant to their own organization or perceptions. The challenge is to find which perceived problems are appropriate for inter-organization problem-solving and which can be solved within individual organizations.

## Background

Mental ill health is among the leading causes of the global health-related burden [1] and affects 10–20% of children and youth worldwide [2]. Children and youth might need care and support to manage their mental ill health, and such care and support may involve various professionals and organizations, depending on such factors as age, severity level and situation. Over the years, the treatment of mental ill health has become more specialized and is provided by an increasing number of departments and organizations [3]. As a result of the specialized treatment and increased number of involved stakeholders, services are often fragmented, inefficient and unco-ordinated [4] with negative consequences for the service user and their family. In addition to this, co-ordination and collaboration are often hindered by different structural frameworks, such as different regulations, legislation as well as differences in organisation and financing [5]. In this study, co-ordination refers to activities within organizations, whereas collaboration is used to denote different types of collaboration between organizations, sometimes mentioned as inter-organizational collaboration.

In Sweden, the challenges of mental ill health among children and youth are similar to those faced by other European countries [6, 7]. Care and support for the group is provided by several different stakeholders, such as Child and Adolescent Psychiatry, Youth Health Clinics, Adult Psychiatry, Local Health Care Centres, Social services, Schools, and Student health. These providers are regulated by different Swedish laws such as the *Swedish Health and Medical Care Act*, the *Swedish School Law*, and the *Swedish Social Services Act*. A lack of co-ordination and collaboration has been identified, especially collaboration between primary care, specialist care, schools and student health [8]. A need for improved co-ordination and collaboration was highlighted in a government-initiated inquiry about how to achieve integrated care of good quality for children and youth [8]. However, there is a lack of knowledge on how to accomplish this.

In research, the advantages of collaboration between different stakeholders are well documented [9–13], and improved collaboration practices and arrangements have been shown to lead to positive consequences with benefits for users, especially groups with multiple problems [9, 14]. Hence, there is great potential in developing a more integrated, collaborative approach to service delivery in the care of children and youth with mental ill health [15]. However, collaboration has barriers. For example, it is difficult to maintain, takes a long time to develop and is resource consuming [13]. Nevertheless, some argue that there is no other way to tackle important, complex issues [12, 13]. Previous research has identified a number of barriers to collaboration [16–18], from administrative/regulative to clinical barriers [16], which need to be considered for successful improvement processes.

An important part of the process to improve collaboration and manage some of the identified barriers is to formulate a shared understanding and a joint problem formulation [19]. To achieve this, a first step is to explore the different stakeholders’ perceived problems. This knowledge can be used to identify relevant improvement activities to reduce fragmentation and improve support and care. To succeed with improved care and support, the involvement of stakeholders other than healthcare providers, such as the service user, the family, social services, schools, and student health, is needed in collaboration. The barriers to collaboration have previously been studied from the perspectives of different professionals [18] and include the allocation of responsibilities, confidence and the professional encounter. There has also been study from the separate perspective of how the service users and families perceive collaboration [20], which has found that structure and trust are important to the development of collaboration. These perspectives add to those brought up by the healthcare professionals. By letting the service users take an active role in identifying problems, additional perspectives can be identified, which could help to improve the care [21, 22].

To the best of our knowledge, no studies have explored the perceived problems of delivering care and support for children and youth with mental ill health from the perspective of all concerned stakeholders. The inclusion of all stakeholders improves the likelihood of achieving a better understanding of the problems, identifying shared understanding and formulating a joint problem. This knowledge could both create better conditions for enhanced collaboration and be used to identify relevant improvement activities to reduce fragmentation, thereby improving care and support for children and youth with mental ill health. Thus, as a contribution to both research and practice, this study aimed to explore perceived problems associated with delivering care and support to children and youth with mental ill health among different stakeholders, and to discuss how the perceived problems relate to collaboration.

## Methods

This study employed a descriptive, qualitative approach based on short statements written by stakeholders involved in the care and support of children and youth with mental ill health. The data collection was embedded in an ongoing development project to promote collaboration concerning mental ill health among children and youth.

### Study setting

The data were collected in the region of Västmanland, which is centrally located in Sweden, and consist of ten municipalities. Both by surface area and population, it is a smaller-sized region. The mental health status in the region does not diverge from the general situation in Sweden [23].

### Participants

A total of 26 participants from all types of concerned stakeholders were included to ensure that all perspectives and experiences were covered, as shown in Table 1. They represented school and student health, primary health care, specialist care, social services, and different service user organizations. The sampling strategy for the workshop was purposeful [24], and the participants selected based on a number of criteria to ensure representation from all relevant stakeholders. The number of participants from each stakeholder varied in the participant group depending on the size and complexity of organizations, geographical location (rural/urban) and also experience of the participants.

**Table 1:** Composition of participant group

Composition of participants	Number
School and student health	9
Primary health care and specialist care	9
Social services	3
Service user organizations	5
Total:	26

### Data collection

Data were collected during a single occurrence, which was an inter-organizational workshop held in February 2020. The data collection method was inspired by rapid appraisal studies, which is an approach used for gaining a quick preliminary, qualitative understanding of a situation [25]. In this case, it was a rapid way to identify the essential elements for making preliminary conclusions used in the forthcoming design and implementation of activities in a development project. The workshop arrangement entailed elements of iterative data collection, where the participants were encouraged to contribute in different steps. A system perspective involving all concerned stakeholders was used to reach a comprehensive perspective of problems perceived by stakeholders in the system.

A workshop with several representatives from each type of stakeholder, conducted in several steps, was chosen to obtain a dynamic interaction between participants. This type of design allowed for identifying problems in several steps individually and collectively to assure that different types of problems and several perspectives were captured. The participants were asked to provide a brief answer to the following question (translated): *What problems related to support and care of children and youth with mental ill health do you perceive?*

Each problem was documented individually on post-it notes in Swedish. The workshop was structured in three steps with the following structure:

*Step 1*: Notes were written individually.

*Step 2*: Notes were discussed in homogeneous stakeholder groups. Additional notes could be added.

*Step 3*: Notes were discussed in mixed stakeholder groups. Additional notes could be added.

**Fig. 1:**
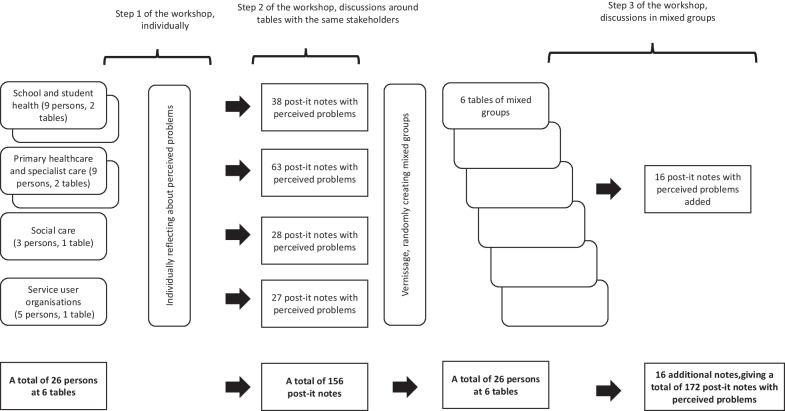
A flowchart of the workshop participants and set-up

The method used for the workshop set-up and process is described in Fig. 1.

After the possibility to add notes in all three steps saturation was assumed to be achieved.

Traceability of stakeholder affiliation in steps 1 and 2 was kept by using different colours on the post-it notes for the different stakeholders. In step 3, all the mixed groups used an additional, uniform colour on the post-it notes.

### Data analysis

Data were analysed using inductive qualitative content analysis [24] with a summative approach [26]. The summative approach implies integrating the number and proportion of statements from different stakeholders in different levels in the analysis to both understand the statement context and at the same time describe the written content.

Each perceived problem was presented on a post-it note in a condensed form, with an average number of 11 words (min = 2, max = 34). As the statements on the notes had such a condensed form, they were used as codes in the content analysis.

In the first step of the analysis, the first author (MH) read the codes repeatedly to get an overall sense of what was predominant in, and characteristic of, the data. In the second step, the codes were analysed and discussed several times by all authors employing an inductive approach, and categories and subcategories were developed [24]. All authors participated in the analysis process, and there was constant movement between the data and the categories.

### Trustworthiness

The analysis was mainly performed by MH and discussed several times with MF and MLK to increase trustworthiness. Reflexive memos of the discussions were collected by MH to maintain an awareness of personal biases or judgements during the process of analysis.

One of the authors (MH) was employed by the region, and hosting the workshop was a part of their role as an improvement leader. However, MH did not have any dependency towards involved stakeholders, which is important both in terms of data collection and data analyses.

### Ethical considerations

The study was reviewed by the Swedish Ethical Review Authority (DNR 2020-06200) and was considered not to be covered by the scope of the Swedish Ethical Review Act, as it does not handle sensitive personal data. All data were handled in accordance with the General Data Protection Regulation (EU 2016/679) and stored in a secure place.

## Results

The analysis included 172 statements in total and was summarized in an inductively produced tentative model of perceived problems concerning the care and support of children and youth with mental ill health.

The model contained the four main categories ***Resources and governance; Collaboration and co-ordination; Knowledge and competence***; and ***Stigma and confidence*** and had 24 subcategories. These categories and subcategories were distributed over three levels: *Societal level, Organizational level* and *Individual level* (see Table 2).

**Table 2:** Levels, categories, and subcategories in the tentative model of perceived problems concerning the care and support of children and youth with mental ill health

	Resources and governance	Collaboration and co-ordination	Knowledge and competence	Stigma and confidence
Societal level	Lack of governance from a national/political perspective Regional differences		Lack of knowledge in society	Stigma in society
Organizational level	Long waiting times Lack of resources Insufficient management Problems with competence acquisition and development	Lack of co-ordination and lack of consensus between organizations Being passed around between stakeholders Troublesome working methods Troublesome sharing of confidential information	Lack of knowledge of each other’s assignments Lack of knowledge within a group/organization	Stigma among professionals Lack of trust between organizations Unprofessional behaviour and denigration
Individual level	Need to fight to get help Lack of support to relatives	Lack of individual perspective	Lack of knowledge about where to seek help	Low trust in adults and care among young people Not utilizing the relatives as resources Negative influence of relatives

The subcategories found in the empirical material are illustrated with examples of the perceived problems, as shown in Table 3.

**Table 3:** Example from the analysis of codes, subcategories, categories and levels

Code	Subcategory	Category	Level
“Short-term political investments are a problem, it is not long-term”	Lack of governance from a national/political perspective	Resources and governance	Societal
“Inequality in access to care in the county”	Regional differences	Resources and governance	Societal
“Long waiting times break down children and families”	Long waiting times	Resources and governance	Organizational
“Many feel they need to fight to get help”	Need to fight to get help	Resources and governance	Individual
“Gap in transitions from, for example, school or social services to specialist care”	Lack of co-ordination and lack of consensus between organizations	Collaboration and co-ordination	Organizational
“Actors in health care are working in silos”	Being passed around between stakeholders	Collaboration and co-ordination	Organizational
“Poor knowledge of each other’s responsibilities”	Lack of knowledge of each other’s assignments	Knowledge and competence	Organizational
“Difficult for parents to know what help is available and how to get there”	Lack of knowledge about where to seek help	Knowledge and competence	Individual
“Fear of talking about mental ill health”	Stigma in society	Stigma and confidence	Societal
“Lack of trust between stakeholders”	Lack of trust between organizations	Stigma and confidence	Organizational
“Why is the power of relatives not used?”	Not utilizing the relatives as resources	Stigma and confidence	Individual

### Resources and governance

The category ***Resources and governance*** (*n* = 59) included eight subcategories distributed over all three levels.

At the societal level, the subcategory ***Lack of governance from a national/political perspective*** included both statements about lack of long-term plans and perspectives for resource management, and lack of national plans and guidelines in the area. This was sometimes expressed in terms of concerns about the difficulty of providing the resources, with one statement reading: *“Where can we find resources when mental ill health increases?” (Primary health care and specialist care).* The subcategory ***Regional differences*** was also at the societal level where one example of this subcategory was unequal access to care and support in the region, exemplified by a statement indicating that it is* “Difficult employing and keeping qualified professionals in more rural parts of the region” (Primary health care and specialist care).*

The organizational level contained four subcategories, including the two subcategories with the largest number of statements in the ***Resources and governance*** category (***Long waiting times*** and ***Lack of resources***). The subcategory ***Long waiting times*** (*n* = 13) included several statements about the perceived problem of waiting times in the care process such as *“Long periods of waiting break children and families. When they ask for help it is needed NOW!” (Service user organizations)*. The large subcategory ***Lack of resources*** (*n* = 15) related to perceived problems concerning resources and a strained system due to lack of resources. Also mentioned in this subcategory was a lack of early and urgent efforts for young children when mental ill health debuts. One such statement read *“Lack of early efforts and co-ordination” (Primary health care and specialist care).* The involved organizations have widely varying missions and regulatory frameworks concerning care and support for children and youth with mental ill health, which was reflected in this category and expressed in the subcategory ***Insufficient management*** with statements illustrating a lack of commitment and understanding. Finally, at the organizational level, there was a subcategory mentioning ***Problems with competence acquisition and development*** as a perceived problem.

There were two subcategories at the individual level. One subcategory, ***Need to fight to get help***, expressed a lack of access to support and care, and a feeling of a need to fight to get help exemplified by the statement: *“It is a problem to get the help you need” (Social services).* The other subcategory was ***Lack of support to relatives*** highlighting a need of support where a statement read *“Unclear what the support for parents should be” (Primary health care and specialist care)*.

### Collaboration and co-ordination

The category ***Collaboration and co-ordination*** (*n* = 73) included five subcategories at the organizational and individual level. The category comprised perceptions of a lack of collaboration and co-ordination in terms of unclear and problematic interfaces between and within the involved stakeholders. This was illustrated by a lack of clarity regarding which level of care should be provided for different severity of mental ill health or failing handovers between actors due to age restrictions. The two subcategories ***Lack of co-ordination and lack of consensus between organizations*** (*n* = 21) and ***Being passed around between stakeholders*** (*n* = 21) showed perception of such problems *between* organizations and *within* an organization. ***Troublesome working methods***, which made co-ordination more difficult, also belonged to this category. Another large subcategory (*n* = 19) was ***Lack of individual perspective***, which referred to perceptions about the failure to listen to the youth and to consider their perspective.

At the organizational level, there were four subcategories. The subcategory ***Lack of co-ordination and lack of consensus between organizations*** gathered perceived problems regarding insufficient co-ordination and a lack of consensus between different organizations. Other perceived problems mentioned as statements were a lack of inter-professional collaboration, complicated contact paths for professionals and a gap in transitions between stakeholders (e.g. social services and specialist care). ***Being passed around between stakeholders*** was perceived as a problem among the stakeholders and was illustrated by a rich variation of examples where one such example was that the service users *“Fall through the cracks*—*too ill for first line care and too healthy for specialist care” (School and student health)*, and another was the risk of discontinuous or lost care when becoming 18 years old and thus being treated as an adult*.* The subcategory ***Troublesome working methods*** reflected concrete perceived problems related to the professionals’ working methods, such as shortages in IT-systems or complicated referral procedures. *“Inflexible transfers of information regarding parental consent” (School and student health)* exemplified perceived problems with sharing confidential information belonging to the subcategory ***Troublesome sharing confidential information***.

This category held one subcategory at the individual level, ***Lack of individual perspective***, where there was a perception of lack of individual perspective in many ways illustrated by statements pointing out a lack of individual perspective in appointments, for example, not including the service users’ preferences enough. In addition, a lack of child and youth perspective when planning the care was mentioned, with concern that this group do not know their rights. One statement read *“Young people are not sufficiently involved; the adults try to solve their problems without asking” (Service user organizations)*.

### Knowledge and competence

The category ***Knowledge and competence*** (*n* = 22) contained four subcategories at all three levels. It gathered perceptions about lack of knowledge and competence within different areas, which included, among other things, ***Lack of knowledge in society*** about mental ill health and ***Lack of knowledge of each other’s assignments***, about the different assignments and missions among involved actors. The category also included ***Lack of knowledge where to seek help*** among the youth.

At the societal level, the subcategory ***Lack of knowledge in society*** mentioned that a problem could be that children and parents have too little knowledge about emotions, feelings and life’s ups and downs. It was also mentioned that it is a problem that the concept of mental ill health has different meanings for different receivers, with one statement reading *“Different view of the concept of mental ill health” (School and student health).*

The organizational level included two subcategories, one of which referred to perceptions about ***Lack of knowledge of each other’s assignments*** which may lead to misunderstandings and lack of comprehension. The other subcategory concerned ***Lack of knowledge within a group/organization*** such as *“Lack of knowledge about mental ill health in school” (Service user organizations)* and *“Lack of knowledge about multicultural differences” (School and student health)*.

One subcategory was at the individual level, ***Lack of knowledge about where to seek help***. That was expressed as *“Hard-to-reach care. The young do not know how to seek care, and even when they do know it is perceived as complicated” (Service user organizations)*. It was also mentioned that it was difficult for parents to know what help that was available and how to find that help.

### Stigma and confidence

***Stigma and confidence*** (*n* = 18) consisted of seven subcategories distributed over all three levels. The category covered perceptions of lack of trust and confidence at different levels, and also perceptions of stigma in terms of prejudice and ignorance at different levels.

The societal level referred to stigma regarding mental ill health that could be perceived in the society and consisted of one subcategory, ***Stigma in society***. It was mentioned in the statements as a general fear of talking about mental ill health and also as a perception of stigma in society.

At the organizational level, the subcategories related to perceived problems about stigma and lack of confidence for organizations and professionals. ***Stigma among professionals*** gathered perceived problems about a fear among the professionals of talking about related issues with children, youth, and relatives. Here one statement read: *“Difficult to dare to talk about, for example, parents’ mental illness” (Social services).* ***Lack of trust between organizations*** referred to perceived problems such as disbelief in the competence of others or a perception of organizations crossing their boundary of responsibility, and that there was no trust between different stakeholders. A subcategory was ***Unprofessional behaviour and denigration***, and a statement that provided a blatant example of this was *“Mudslinging of other stakeholders in front of parents” (Social services).*

At the individual level, three subcategories could be found: one about ***Low trust in adults and care among young people***; and two subcategories concerning the role of relatives, ***Not utilizing the relatives as resources*** and ***Negative influence of relatives***. There were perceived problems concerning service users such as a pupil, a patient, a user or a relative having a low level of trust in health care, support from other professionals, and in adults. This was expressed as adults in general not listening carefully enough. For the subcategory ***Not utilizing the relatives as resources***, one issue that was raised was that relatives are not used as a possible resource in the care. Finally, the subcategory ***Negative influence of relatives*** related to relatives obstructing or failing to appear and included a statement *“Problems when parents obstruct and do not consider what is best for the children” (Primary health care and specialist care)*.

### Perceived problems divided by stakeholder

The different groups of stakeholders reported perceived problems in different areas. In six subcategories, however, all four stakeholder groups noticed perceived problems, five subcategories at the organizational level and one at the individual level, as shown in Table 4. The stakeholder groups shared the perception that problems were: ***Long waiting times***, a ***Lack of resources***, that service users ***Need to fight to get help***, a ***Lack of co-ordination and lack of consensus between organizations, Being passed around between stakeholders*** and a ***Lack of knowledge within a group/organization***. All other subcategories with perceived problems were highlighted by one or several stakeholder groups, but not shared by all.

**Table 4:** The subcategories that all involved stakeholder groups perceived as problems

Subcategory	Category	Level
Long waiting times	Resources and governance	Organizational level
Lack of resources	Resources and governance	Organizational level
Need to fight to get help	Resources and governance	Individual level
Lack of co-ordination and lack of consensus between organizations	Collaboration and co-ordination	Organizational level
Being passed around between stakeholders	Collaboration and co-ordination	Organizational level
Lack of knowledge within a group/organization	Knowledge and competence	Organizational level

A presentation of all the stakeholders’ reported perceived problems in this study is shown in Table 5. In eleven of the subcategories, three or four stakeholder groups stated perceived problems, and in five subcategories just one stakeholder specified perceived problems. This indicates that the stakeholders only have a partially shared understanding and joint problem formulation.

**Table 5:** A total account of the different stakeholder groups reported perceived problems

Category	Subcategory	Stakeholder			
		S1	S2	S3	S4
*Societal level*					
Resources and governance	Lack of governance from a national/political perspective		X		
	Regional differences	X	X		
Knowledge and competence	Lack of knowledge in society	X			
Stigma and confidence	Stigma in society		X		X
*Organizational level*					
Resources and governance	Long waiting times	X	X	X	X
	Lack of resources	X	X	X	X
	Insufficient management	X	X	X	
	Problems with competence acquisition and development		X		
Collaboration and co-ordination	Lack of co-ordination and lack of consensus between organizations	X	X	X	X
	Being passed around between stakeholders	X	X	X	X
	Troublesome working methods	X	X	X	
	Troublesome sharing confidential information	X	X		
Knowledge and competence	Lack of knowledge of each other’s assignment	X	X	X	
	Lack of knowledge within a group/organization	X	X	X	X
Stigma and confidence	Stigma among professionals		X	X	
	Lack of trust between organizations		X	X	
	Unprofessional behaviour and denigration			X	
*Individual level*					
Resources and governance	Need to fight to get help	X	X	X	X
	Lack of support to relatives		X	X	
Collaboration and co-ordination	Lack of individual perspective		X	X	X
Knowledge and competence	Lack of knowledge about where to seek help	X	X		X
Stigma and confidence	Low trust in adults and care among young people				X
	Not utilizing the relatives as resources	X	X		
	Negative influence of relatives	X	X		

S1, School and student health; S2, Primary health care and specialist care; S3, Social services; S4, Service user organizations

The distribution of the different stakeholder group statements in the four different categories varied. For three of the stakeholder groups, the most represented category was ***Collaboration and co-ordination*** and ***Resources and governance*** was also a quantitatively large category.

The distribution of statements differed among the stakeholder groups between the three levels. The *Service user organizations* were more prone to report statements at the individual level than other stakeholders. The statements of the other three stakeholders, *School and student health, Primary health care and specialist care* and *Social services,* were more focused at the organizational level. Only a smaller part of all statements corresponded to the societal level.

## Discussion

The aim of this study was to explore perceived problems associated with delivering support and care to children and youth with mental ill health, and to discuss how the perceived problems relate to collaboration. The perceived problems were summarized in a model consisting of four main categories: ***Resources and governance***; ***Collaboration and co-ordination***; ***Knowledge and competence***; and ***Stigma and confidence***, and 24 subcategories. These categories and subcategories were distributed over three levels: *Societal level, Organizational level* and *Individual level*, indicating that the perceived problems are complex, targeting several areas and system levels, sometimes in combination*.* A majority of the perceived problems were related to the organizational level, and just a minor proportion to the societal level. Overall, all involved stakeholder groups perceived problems in all four main categories. Six of the subcategories were shared by all stakeholder groups: for example, ***Long waiting times*** and ***Lack of co-ordination and lack of consensus between organizations***. Five out of these six shared subcategories were found at the organizational level.

The perceived problems identified in this study are similar to findings in previous studies [17, 18, 20, 27]; for example, the subcategories ***Need to fight to get help*** [20, 27], ***Lack of co-ordination and lack of consensus between organizations*** [17, 18, 20] and ***Lack of knowledge about where to seek help*** [17, 18, 20, 27]. Using corresponding concepts, the findings from other studies have been discussed as barriers related to either collaboration or assessing care and support for children and youth with mental ill health.

On an overall level, the stakeholder groups shared perceptions of problems related to care and support for children and youth with mental ill health. However, when further explored in subcategories, a variation in the type of problems highlighted by different stakeholder groups emerged. This does not necessarily imply that a stakeholder group disagreed with the problems identified by another stakeholder. Instead, it could be interpreted as these problems not being perceived as the most relevant and urgent for that particular stakeholder at that time.

The importance of a shared understanding and joint problem formulation has been highlighted as an important part of the process to improve collaboration and manage some of the identified barriers [19]. In this study, the findings indicate that the stakeholders do not have a completely shared understanding of the perceived problems that could affect the ability to improve collaboration. Another finding was that stakeholders were more prone to report perceived problems closely related to themselves and their organizations. For example, the service user organizations to a larger extent reported problems on the individual level, whereas stakeholder groups that provide care and support mainly reported problems related to the organizational level. This might be a challenge, or a barrier, in the effort to obtain a joint problem formulation. Despite this challenge, attendance of different stakeholders in improvement efforts seems to be important to reflect upon as lack of relevant stakeholders could result in a loss of essential aspects of perceived problems related to care and support for this group. This, in turn, could lead to decisions on actions being taken on an incorrect basis, which could have consequences for one or more of the actors involved. Involving the service users in improvement efforts seems to add a different perspective than brought up by the other stakeholder groups. The recommendation to involve patients or service users has also been mentioned by others [21, 22].

One part of the findings in the present study was perceived problems related to ***Stigma and confidence***. The category occurred at all levels (societal, organizational, and individual). Some aspects, such as the subcategory ***Stigma in society***, might be difficult for the involved stakeholders to address, but still influence the prerequisite for care and support. Such aspects are important to consider in improvement processes, even if they are not directly targeted.

### Perceived problems with care and support in relation to collaboration

A number of different stakeholders need to be involved to make care and support work in this area, and the need for collaboration to achieve co-ordinated care of good quality for children and youth has been mentioned earlier. A considerable proportion of the perceived problems in the current study is directly or indirectly related to collaboration. The category ***Collaboration and co-ordination*** gathers perceived problems concerning the fulfilment of collaboration and co-ordination between and within different stakeholders. The remaining three categories relate to collaboration by dealing with prerequisites to achieve collaboration and are similar to previously identified barriers (i.e. factors that characterize unsuccessful collaboration) [18].

Collaboration has been described as resource demanding and difficult to maintain [13], but necessary to tackle important, complex issues such as those in this area. Some of the needs for well-functioning collaboration (e.g. resources) are mentioned as a shortage in the present study, a perceived problem that was shared by all stakeholder groups. However, as collaboration is difficult, takes time and is unstable [28], perhaps not all problems should be solved by collaboration. This study shows perceived problems identified by different stakeholder groups, but these should not be confused with who needs to be involved in addressing the issues. Some problems may only need one organization to manage them, while others may require the involvement of two or more stakeholders. Therefore, it is important to investigate which problems require a broader collaboration involving several stakeholders and their resources, and which could be solved in other ways. Even though care and support is complex and requires collaboration [12, 13], the approaches still need to be balanced against each other to find the most appropriate way given the resources required and the desired results. Instead of seeking a shared and joint problem formulation among all stakeholders, it might be sufficient to be aware of perceived problems affecting joint service users in adjoining parts of care and support. Knowledge of the skills and working conditions of other stakeholders involved, as well as respect and mutual trust among the collaborators, could provide an increased awareness of the problems of others and have been found to promote collaboration over time [29].

### Strengths and limitations

This qualitative study has several strengths. First, the participants in the study represent the stakeholder groups involved in the care and support of children and youth with mental ill health well. Second, compared with previous work [18, 30], the current study includes a wider range of stakeholders. In addition to organizational stakeholders representing health care, social services, and school, this study also includes representatives from service user organizations to cover the perspective of children, youth, and relatives. This perspective has been studied separately previously [20]. In this study, the inclusion of the service user organizations provides an additional perspective, and in numbers they created a larger share of statements at the individual level than the other stakeholder groups. Third, the data collection and transcription were performed by the same researcher, ensuring that the method was followed, and no data were lost. Fourth, the participants wrote statements of individual perceived problems, as well as discussing perceived problems around the table, and contributed with further statements of perceived problems from the discussions. Finally, trustworthiness was addressed by involving several researchers with experience of qualitative analysis in the analysis process.

The study also has some limitations. It might be seen as a limitation that the data collection took place on a single occasion with a limited number of participants. This should be kept in mind when interpreting the results, especially regarding the subgroup analysis. However, the selection of participants to achieve broad representation was carried out thoroughly, and the workshop where data were collected was accomplished at a pleasant pace without stress. Another limitation could involve conducting qualitative analyses on statements that have a condensed and concise form, which may have impeded a deeper understanding. To mitigate this, the statement’s text was used verbatim throughout the analysis. Another limitation could be the limited geographical spread, as all participants are linked to the county of Västmanland in Sweden. Finally, the participants were asked to reflect upon perceived problems with the care and support of children and youth with mental ill health, but to leave out positive examples of care and support. Identifying the positive examples and being aware of those may have been important to make sure that successful parts of collaboration or processes were maintained before decisions were made on improvement activities.

To deepen the understanding of problems in this area and to investigate the composition of a joint problem formulation, further studies are needed. A possible area of future studies is how to proceed the work process after identifying perceived problems and to investigate which problems require a collaborative approach.

## Conclusions

The results from this study show that the perceived problems relating to the care and support of children and youth with mental ill health were either directly or indirectly related to collaboration or co-ordination, often acting as barriers to achieve successful collaboration. The problems were distributed on several levels, indicating a complex scenario that requires collaboration to be solved. Even though the perceived problems were shared by stakeholders on an overall level, the findings indicate that the various stakeholder groups do not have a completely shared understanding of the perceived problems associated with support and care of children and youth with mental ill health. The reported problems ranged from lack of national governance, related to the structure of the system, to how to involve relatives in the individual situation and were often closely related to the stakeholder’s perspective. That indicates the need to include a wide range of stakeholders in the process to identify the breadth of the problems relevant to the area. To gather all involved stakeholders to share with each other and discuss the perceptions of problems from different perspectives during the problem identification phase is a valuable and efficient approach for achieving a comprehensive view of important perceived problems. Especially in improvement processes within complex areas where activities may need to target, for example, collaborative structures at different levels, the functionality of the service system as well as the support that children, youth and their relatives receive in individual situations. Importantly, this study shows perceived problems identified by different stakeholders, although these should not be confused with the stakeholders that need to be involved to *solve* the problems. Some perceived problems are probably best solved by one or a few of the stakeholders, whereas others related to the functionality of the service system may require wider participation. It is suggested that large complex areas involving several stakeholders from various organizations might not require a joint problem formulation to be able to improve collaboration—sometimes a shared picture of the perceived problems may be enough. It is also important to investigate which problems require broader collaboration involving several stakeholders and resources, and which ones may be solved in other ways.

## Data Availability

The dataset used and analysed during the current study is available in Swedish from the corresponding author on reasonable request.
